# The atypical chemokine receptor-2 fine-tunes the immune response in herpes stromal keratitis

**DOI:** 10.3389/fimmu.2022.1054260

**Published:** 2022-11-28

**Authors:** Tian Yu, Fabian Schuette, Maria Christofi, John V. Forrester, Gerard J. Graham, Lucia Kuffova

**Affiliations:** ^1^ Division of Applied Medicine, Section of Immunity, Infection and Inflammation (Ocular Immunology), Institute of Medical Sciences, University of Aberdeen, Aberdeen, United Kingdom; ^2^ Department of Ophthalmology, Beijing Hospital, National Center of Gerontology, Beijing, China; ^3^ Chemokine Research Group, Institute of Infection, Immunity and Inflammation, College of Medical, Veterinary and Life Sciences, University of Glasgow, Glasgow, United Kingdom; ^4^ Ocular Immunology Program, Centre for Ophthalmology and Visual Science, The University of Western Australia, Perth, WA, Australia; ^5^ Centre for Experimental Immunology, Lions Eye Institute, Perth, WA, Australia; ^6^ Eye Clinic, Aberdeen Royal Infirmary, Aberdeen, United Kingdom

**Keywords:** atypical chemokine receptor-2, herpes stromal keratitis, antigen presenting cell, adaptive immunity, neovascularization

## Abstract

Herpes stromal keratitis (HSK) is a blinding corneal disease caused by herpes simplex virus-1 (HSV-1), a common pathogen infecting most of the world’s population. Inflammation in HSK is chemokine-dependent, particularly CXCL10 and less so the CC chemokines. The atypical chemokine receptor-2 (ACKR2) is a decoy receptor predominantly for pro-inflammatory CC chemokines, which regulates the inflammatory response by scavenging inflammatory chemokines thereby modulating leukocyte infiltration. Deletion of ACKR2 exacerbates and delays the resolution of the inflammatory response in most models. ACKR2 also regulates lymphangiogenesis and mammary duct development through the recruitment of tissue-remodeling macrophages. Here, we demonstrate a dose-dependent upregulation of ACKR2 during corneal HSV-1 infection. At an HSV inoculum dose of 5.4 x 10^5^ pfu, but not at higher dose, ACKR2 deficient mice showed prolonged clinical signs of HSK, increased infiltration of leukocytes and persistent corneal neovascularization. Viral clearance and T cell activation were similar in ACKR2^-/-^ and wild type mice, despite a transient diminished expression of CD40 and CD86 in dendritic cells. The data suggest that ACKR2 fine-tunes the inflammatory response and the level of neovascularization in the HSK.

## Introduction

Corneal blindness and visual impairment (BVI) accounts for 2.4% of the globally estimated 253 million visually impaired people (1). Herpes simplex infection of the cornea is a common cause of corneal BVI with an incidence of up to ~35.0 per 100,000 person-years [reviewed in ref (2)]. Primary infection of the corneal epithelium leads to a transient replication of virus, spread to sub-epithelial sensory nerves and trafficking of virus to the trigeminal ganglion (TG) where it establishes life-long latency (2). Induction of a corneal innate immune response (infiltrating inflammatory macrophages, natural killer (NK) cells and neutrophils) promotes viral clearance (3–5) while antigen presenting cells (APCs) endocytose viral fragments and migrate to the draining lymph node (DLN), where they induce a predominantly CD4^+^ T response (2, 6, 7). This leads to immune mediated pathology of the corneal tissue resulting in herpes stromal keratitis (HSK) (2, 8). HSK is characterized by corneal opacification, ulceration, oedema and neovascularization during the active phase and can result in corneal scarring and permanent loss of vision (2, 9). Restoration of vision is only possible *via* corneal transplantation, but rejection rates and early graft failure are high due to corneal vascularization and possible reactivation of virus (10, 11). Thus, understanding the immunopathogenesis of HSK is of important potential therapeutic significance.

The atypical chemokine receptor-2 (ACKR2) is a chemokine decoy receptor that binds to and promiscuously scavenges pro-inflammatory, but not homeostatic, CC chemokines (12). By competing with conventional chemokine receptors, it thus reduces their pro-inflammatory impact since signaling *via* chemokines is disrupted. ACKR2 thus plays a modulating role during inflammation. ACKR2 is strongly expressed by lymphatic endothelial cells (LECs), trophoblasts and, as recently shown, lung capillary vascular endothelial cells (12–14), as well as subsets of leukocytes including innate-like B cells, dendritic cells (DCs), macrophages and monocytes (12, 15). In the absence of ACKR2, inflammatory responses appear unregulated with increased levels of chemokines, leukocyte infiltration and augmented fibrosis (16–20). Moreover, the expression of ACKR2 on LECs has been shown to facilitate lymph flow and APC migration *via* elimination of excessive pro-inflammatory chemokines (21, 22).

Previously, ACKR2 has been demonstrated to regulate branching of lymphangiogenic vessels and mammary gland ductules during development by coordinating the migration of tissue-remodeling macrophages (23, 24). Recently, the mechanism of ACKR2 involvement in the development of mammary gland has been further linked to the regulation of CCL7 and subsequently orchestration of CD206^+^ macrophages (25). Furthermore, we have also shown that while ACKR2 played no role in murine corneal allograft rejection, syngeneic corneal grafts (effectively a corneal wound healing model) in ACKR2 deficient mice presented with greater numbers of single Lyve-1^+^cells around lymphatic vessels and accelerated corneal lymphangiogenesis (26). We therefore investigated the role of ACKR2 in corneal angiogenesis in the HSK murine model in which both blood and lymphatic new vessel formation are prominent pathologies.

Here, we show that expression of ACKR2 is upregulated in the corneas after HSV-1 infection. Deletion of ACKR2 leads to prolonged HSK compared to wild type (WT) mice with increased corneal leukocyte infiltration and persistent growth of corneal new vessels, whereas viral clearance in the cornea was not affected. Furthermore, although ACKR2^-/-^ mice displayed reduced DC maturation in the DLN, T cell priming remained at similar levels between ACKR2^-/-^ and WT mice. Thus, ACKR2 limits corneal inflammation during HSK by fine-tuning the local inflammatory response. However, this fine-tuning effect is lost when HSK is induced with a high dose of virus.

## Results

### Low dose HSV-1 induces high levels of ACKR2 expression in the cornea compared to high dose HSV-1 while clinical disease severity correlates inversely with the strength of the HSV-1 challenge in ACKR2^-/-^ vs. WT mice

**Figure 1:**
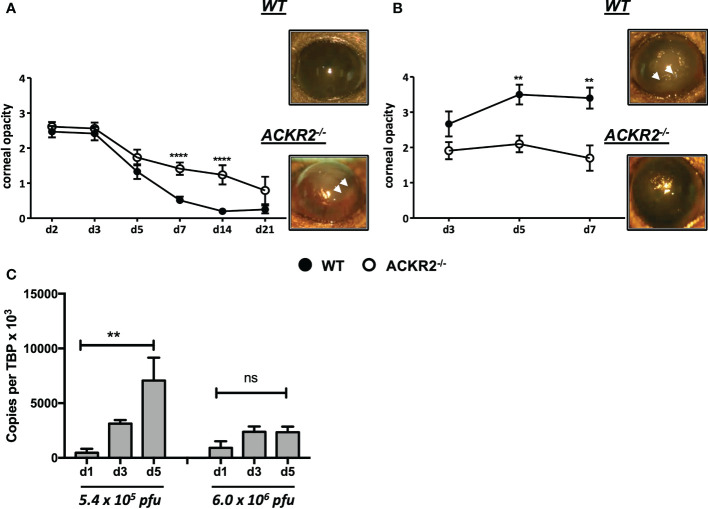
Deletion of ACKR2 exacerbates moderately severe HSK but has no effect on severe/maximal HSK. **(A, B)** Corneas of 6-8 weeks old sex-matched WT (filled circle) and ACKR2^-/-^ (clear circle) mice were infected with HSV-1 virus after corneal scarification. Severity of clinical disease in infected mice was evaluated by corneal opacity scores. Corneal opacity score and representative clinical images at day 7 p.i. in mice infected with **(A)** 5.4 x 10^5^ pfu, N = 6-43; and **(B)** 6.0 x 10^6^ pfu, N = 10-14. White arrow heads indicated level of hypopyon – see main text for explanation. Statistical significance was determined using Mann-Whitney *U* test with **p<0.01, ****p<0.0001. Data are pooled results from independent set of experiments presented as mean ± SEM. **(C)** Corneas from 5.4 x 10^5^ pfu and 6.0 x 10^6^ pfu HSV-1 infected WT mice were harvested at day 1, 3 and 5 p.i. for qPCR analysis of ACKR2 expression. N = 4-6. Statistical significance was determined using one-way ANOVA with **p < 0.01. ns: not significant. Data are presented as mean ± SEM.

The effect of ACKR2 deletion was evaluated using the murine HSK model. Two doses - 5.4 x 10^5^ pfu and 6.0 x 10^6^ pfu of HSV-1 were used to infect the corneas of ACKR2^-/-^ and WT mice. With both doses, ACKR2^-/-^ and WT mice developed significant corneal opacity within 3 days post infection (p.i.) (**Figures 1A, B**). With 5.4 x 10^5^ pfu HSV-1, corneal opacity in the WT mice rapidly resolved at 1week p.i. with near complete restoration of corneal clarity, whereas in ACKR2^-/-^ mice, HSK was significantly prolonged with higher corneal opacity scores at day 7 and 14 p.i. (**Figure 1A**). Furthermore, 5.4 x 10^5^ pfu HSV-1 infection in ACKR2^-/-^ mice was occasionally associated with hypopyon (a collection of inflammatory cells which sediments in the lower anterior chamber, **Figure 1A**, white arrow heads), which was rarely observed in WT mice. With 6.0 x 10^6^ pfu HSV-1 infection, WT mice developed significantly more severe and persistent corneal inflammation compared to mice infected with 5.4 x 10^5^ pfu (**Figures 1A, B**). In addition, lack of ACKR2 did not worsen HSK after infection with 6.0 x 10^6^ pfu of HSV-1 virus which was close to maximal levels in WT mice, and less but still severe in ACKR2^-/-^ mice (**Figure 1B**). Interestingly, despite significant corneal disease, ACKR2^-/-^ mice did not develop hypopyon with 6.0 x 10^6^ pfu HSV-1 unlike WT mice (7 of 10 mice) (**Figure 1B**, white arrowheads).

Since ACKR2 is expressed on a range of cell types including innate-like B cells, subsets of myeloid cells, blood and lymphatic endothelial cells (12, 13), we asked whether ACKR2 is also expressed in corneal tissue. Accordingly, corneas from HSV-1 infected WT and ACKR2^-/-^ mice were harvested for qPCR to test the expression of ACKR2. ACKR2 is minimally expressed in uninfected corneas (data not shown). Interestingly, in 5.4 x 10^5^ pfu HSV-1 infected WT corneas, expression of ACKR2 increased progressively during the course of disease as corneal inflammation resolved, whereas with 6.0 x 10^6^ pfu, no upregulation of ACKR2 was observed (**Figure 1C**). In both groups, ACKR2^-/-^ mice expressed no or negligible levels of ACKR2 (data not shown). These results support a regulatory role for ACKR2 when inflammation is moderate which is lost when inflammation is severe. We therefore further explored the regulatory role of ACKR2 in HSV-1 infected corneas using 5.4 x 10^5^ pfu.

### Leukocyte infiltration in the cornea correlates with clinical phenotype of HSK in ACKR2^-/-^ and WT mice

**Figure 2:**
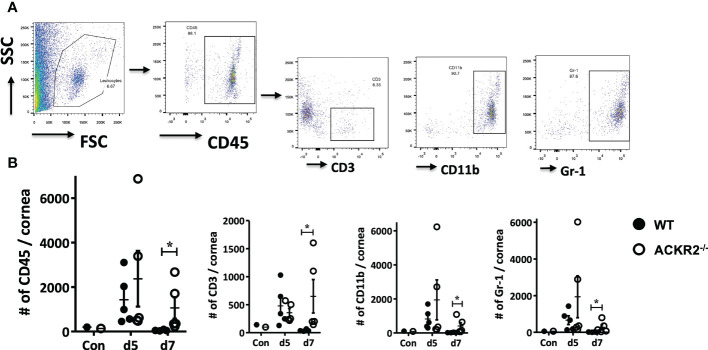
Leukocyte infiltration is increased in ACKR2^-/-^ HSV-1 infected corneas. HSV-1 infected corneas were harvested from ACKR2^-/-^ and WT mice post corneal infection and stained with CD45, CD3, CD11b and Gr-1 for flow cytometry analysis. **(A)** Gating strategy for corneal leukocyte infiltration. CD3, CD11b and Gr-1 subpopulations were gated under CD45 positive population. **(B)** Leukocyte infiltration in infected corneas of ACKR2^-/-^ (clear circle) and WT (filled circle) mice at day 5 and day 7 p.i. Control corneas were collected from untreated ACKR2^-/-^ and WT mice. N = 4-5. Statistical analysis performed with Mann-Whitney *U* test with *p < 0.05. Data are representative of two independent sets of experiments shown as mean ± SEM.

HSK in mice is characterized by early infiltration of innate immune cells into the cornea with later CD4 T cell mediated damage (2). We therefore first evaluated the effect of ACKR2 deficiency on leukocyte infiltration in corneas infected with 5.4 x 10^5^ pfu by flow cytometry (**Figure 2A**). Corneas from uninfected ACKR2^-/-^ and WT mice were used to control resident corneal myeloid cells. ACKR2^-/-^ and WT mice had similar levels of leukocyte infiltration at day 5 p.i. whereas greater numbers of CD45^+^, CD3^+^, CD11b^+^ and Gr-1^+^ cells were observed in ACKR2^-/-^ mice at day 7 p.i. (**Figure 2B**). Interestingly, this enhanced influx of leukocytes in the infected corneas of ACKR2^-/-^ mice was observed across all cell populations studied indicating a general elevated inflammatory status of the ACKR2^-/-^ corneas. Consistent with the clinical signs, infiltration of immune cells persisted in ACKR2^-/-^ mice after corneal HSV-1 infection.

### Viral clearance occurs at a similar tempo in ACKR2^-/-^ and WT mice

**Figure 3:**
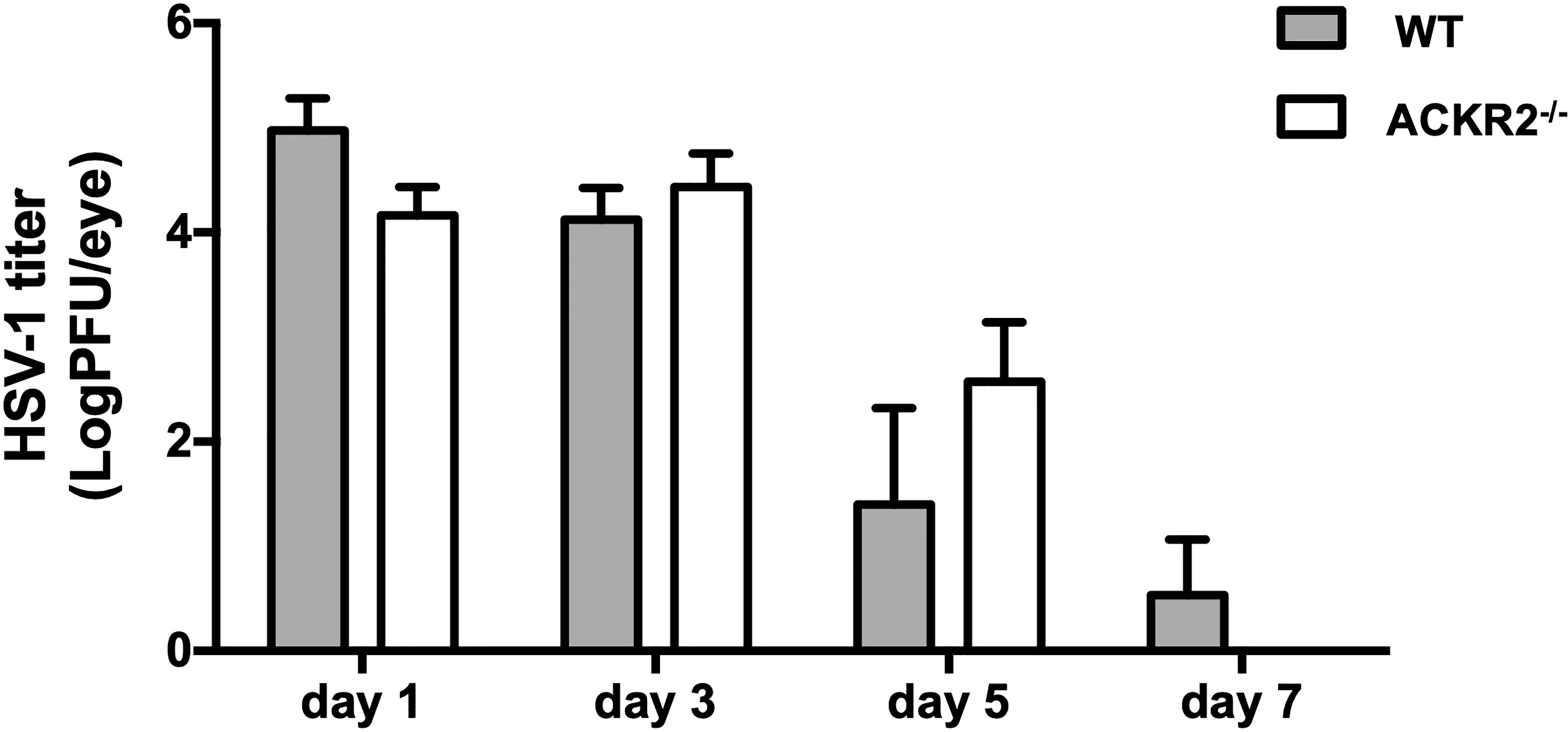
HSV-1 viral burden in corneal tear films of infected mice is similar in ACKR2^-/-^ and WT mice. ACKR2^-/-^ (clear bar) and WT (filled bar) mice were infected with 5.4 x 10^5^ pfu of HSV-1 virus on scarified corneas. Corneal swabs of tear film samples from infected corneas were harvested and snap frozen at day 1, day 3, day 5 and day 7 p.i. and stored in DMEM at -80°C until use. Standard plaque assay was then performed with tear film samples with each dilution plated in triplicates and viral load was calculated for each individual mouse. N = 5. Statistical analysis was performed with unpaired student’s *t* test. Data are representative of two independent experiments and shown as mean ± SEM.

After corneal HSV-1 infection, the virus is usually eliminated from the cornea within 1 week, while virus which has migrated to the TG during this time converts to latency (2). In order to exclude the possibility that prolonged clinical disease and leukocyte infiltration in ACKR2^-/-^ mice might be due to persistent cytopathic virus, plaque assay was performed with tear film samples from ACKR2^-/-^ and WT mice at day 1, 3, 5 and 7 after corneal infection. In both ACKR2^-/-^ and WT mice, virus was effectively cleared from the tear film by day 7 p.i. with no significant difference detected between the two strains of mice (**Figure 3**). This result indicates that viral cytopathic effect was not the cause for prolonged severity of HSK in ACKR2^-/-^ mice.

### Deletion of ACKR2 leads to prolonged growth of corneal neovascularization, but has no effect on early lymphangiogenesis

**Figure 4:**
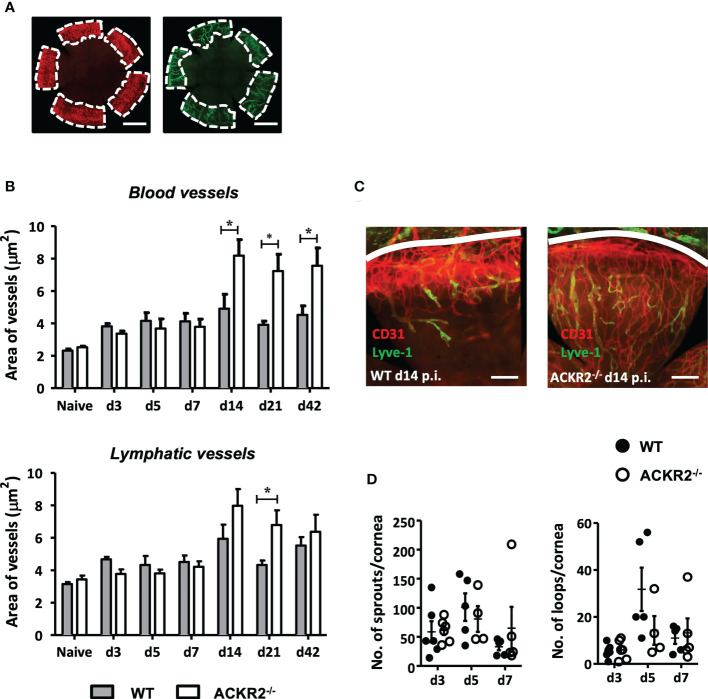
Prolonged development of corneal neovascularization but not lymphangiogenesis in ACKR2^-/-^ mice with HSK. Corneal whole mounts were prepared from ACKR2^-/-^ and WT mice infected with 5.4 x 10^5^ pfu of HSV-1 virus and stained with anti-CD31 (red) and anti-Lyve-1 (green) antibodies for blood and lymphatic vessels respectively. **(A)** Diagram showing region of vessel quantification. Area of vessel invasion indicated by the area of cornea inside dotted white line which is determined by the outer limbal vessel arch and the junction of the inner most blood and lymphatic vessels. Scale bar represents 1000 μm. **(B)** Quantification of area of blood vessel and lymphatic vessel invasion in infected ACKR2^-/-^ (clear bar) and WT (filled bar) mice. Corneas from untreated ACKR2^-/-^ and WT mice were used as naïve controls. N = 4-11. Statistical analysis performed by unpaired student’s *t* test with *p < 0.05. Data are pooled results from independent set of experiments and shown as mean ± SEM. **(C)** Representative corneal whole mount staining at day 14 p.i. stained for CD31 and Lyve-1. White line indicates limbal vessel arcade. Scale bar represents 200 μm. **(D)** Quantification of early lymphangiogenesis in infected corneas of ACKR2-/- (clear circle) and WT (filled circle) mice was performed by numbers of lymphatic sprouts and loops in ImageJ software using the plug-in Lymphatic Vessel Analysis Protocol (LVAP). N = 4-6. Statistical analysis performed by unpaired student’s *t* test. Data are pooled results from three independent experiments and shown as mean ± SEM.

ACKR2 is expressed on leukocytes and lymphatic vessels as well as recently identified on pulmonary vascular endothelial cells (12, 13), but its precise role in the orchestration of the inflammatory response in terms of the vascular vs. the leukocytic response is not clear. Each may be regulated separately, or one may be the consequence of the other, since leukocytes are known to secrete pro-angiogenic factors while vessels facilitate the ingress and egress of leukocytes. ACKR2 has been shown to play a regulatory role during embryonic development of lymphatic vessels, as well as playing a role in a similar phenomenon, i.e., branching morphogenesis of developing mammary gland ducts (23, 24). In the cornea, administration of an ACKR2 mimic “trap” was reported to suppress neovascularization induced by corneal allografts (27). We therefore explored further the role of ACKR2 on HSV-1 induced corneal neovascularization, evaluating both blood and lymphatic vessels. Whole mounts of HSV-1 (5.4 x 10^5^ pfu) infected corneas were prepared and stained with anti-CD31 and anti-Lyve-1 antibodies for quantification of corneal blood and lymphatic vessels respectively (**Figure 4A**). Naïve corneas from ACKR2^-/-^ and WT mice served as controls for baseline quantification of limbal blood and lymphatic vessels. Our data show that, despite the increased severity of HSK in ACKR2^-/-^ mice compared to WT mice at day 7 p.i. (**Figure 1A**), areas of corneal neovascularization showed no difference between the two strains during the first week p.i. (**Figure 4B**). Thereafter, ACKR2^-/-^ mice demonstrated significantly enhanced growth of blood vessels compared to WT mice, which persisted to day 42 p.i. (**Figures 4B, C** **)**. Lymphatic vessels behaved similarly, having developed faster by day 21 p.i. in ACKR2^-/-^ mice compared to WT mice, but unlike blood vessels, the accelerated rate of growth did not persist to day 42 p.i. (**Figures 4B, C** **)**. Moreover, in order to explore the effect of ACKR2 deletion on early lymphangiogenesis, further analysis was performed by quantification of lymphatic sprouts and loops since these are considered the nascent foci of new vessel growth (28). However, there was no difference in these morphological features between ACKR2^-/-^ and WT mice at day 3, 5 and 7 p.i. (**Figure 4D**). Therefore, ACKR2 does not seem to have a direct effect on early corneal lymphangiogenesis induced by HSV-1 infection, but may exert constraints on neovascularization during later stages of HSK.

### Deletion of ACKR2 results in reduced maturation of DC, but does not alter T cell activation in the DLN

**Figure 5:**
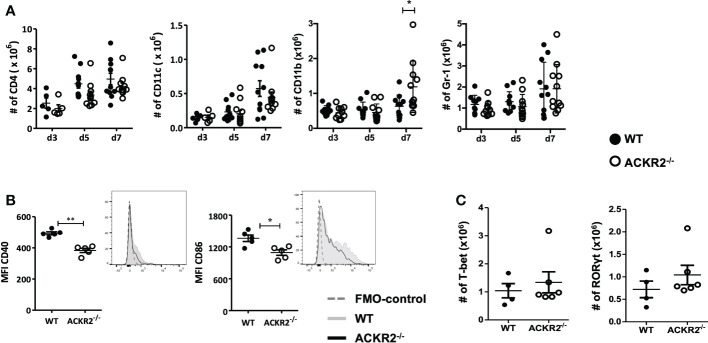
Leukocyte infiltration in the submandibular lymph node. Eye draining submandibular lymph nodes were harvested from HSV-1 infected ACKR2^-/-^ (clear circle) and WT (filled circle) mice for flow cytometry analysis. **(A)** Absolute numbers of leukocyte infiltration in the submandibular lymph node at day 3, day 5 and day 7 p.i. in ACKR2^-/-^ and WT mice. CD4 positive cells were gated under CD3 positive population, CD11b and Gr-1 positive cells were gated under CD45 positive population. N = 5-11. Data were acquired from pooled results of two independent set of experiments. Statistical significance determined by unpaired student’s *t* test. *p<0.05 and data presented as mean ± SEM. **(B)** Mean fluorescence intensity (MFI) calculated by geometric mean for CD40 and CD86 within the CD11c population at day 3 p.i. in mice infected with HSV-1 virus, N = 5. **(C)** Absolute numbers of CD4^+^ T-bet and RORγt cells at day 3 p.i. in ACKR2^-/-^ and WT mice infected with HSV-1 virus. N = 4-6. **(B, C)** Data are representative results of two independent experiments. Statistical significance determined by unpaired student’s *t* test. *p < 0.05, **p < 0.01 and data presented as mean ± SEM.

The adaptive immune response plays a central role during the development of HSK. Following HSV-1 infection, an antigen specific CD4^+^ T cell response is induced in the DLN (2, 8). ACKR2 expression by LECs has been proposed to facilitate efficient migration of APCs towards the DLN (21). We therefore explored immune cell populations in the eye-draining lymph node after corneal infection with 5.4 x10^5^ pfu HSV-1 in ACKR2^-/-^ and WT mice. Our results show that in both ACKR2^-/-^ and WT mice, there was a progressive increase in all leukocyte populations during the first week of infection, but no difference between ACKR2^-/-^ and WT mice in the numbers of CD4^+^, CD11c^+^ and Gr-1^+^ cells (**Figure 5A**). At day 7 p.i. the numbers of CD11b^+^ cells in the DLN were significantly higher in the ACKR2^-/-^ mice compared to WT mice (**Figure 5A**), consistent with the clinical signs at this time (**Figure 1A**). To specifically evaluate DC activation, we examined the expression of co-stimulatory markers CD40 and CD86 among CD11c^+^ cells. Interestingly, ACKR2^-/-^ mice demonstrated reduced expression of both CD40 and CD86 on DCs compared to WT mice at day 3 p.i. (**Figure 5B**), but not at later times (data not shown). However, the transient reduction in DC maturation in ACKR2^-/-^ mice did not translate to a reduced number of T-bet^+^ and RORγt^+^ cells among CD4^+^ T cells (**Figure 5C**). Therefore, our data suggest that T cell activation was not compromised in HSV-1 infected ACKR2^-/-^ mice.

## Discussion

The role of ACKR2 in the inflammatory response has been investigated in several animal models, including chemical- and bacterial- induced inflammation, autoimmune disease and alloimmune disease, but with limited exploration in viral infection (16, 26, 29–32). Contradictory findings have been reported in both experimental autoimmune encephalomyelitis and experimental colitis where opposing disease phenotypes were reported in mice with deletion of ACKR2 (29, 30, 33, 34). Here, we report that the expression of ACKR2 was upregulated in the corneas of WT mice infected with 5.4 x 10^5^ pfu of HSV-1 virus per cornea. Furthermore, in ACKR2 deficient mice, resolution of HSK was delayed as shown clinically by persistence of corneal opacity and by increased leukocyte infiltration. However, when the corneas were infected with a higher viral load of 6.0 x 10^6^ pfu per cornea, ACKR2 failed to upregulate indicating that ACKR2 expression was prevented when inflammation was severe. A similar dose effect has been shown in the lung where ACKR2 reduced the levels of CCL17 and CCL22 during allergic inflammation, but only within a defined concentration range (35). Thus, in the present study any fine-tuned regulatory effect of ACKR2 on HSK was lost at a high viral dose. These data are relevant to the interpretation of conflicting data from previous studies cited above.

We therefore explored further the regulatory role of ACKR2 in HSK using an HSV-1 dose of 5.4 x 10^5^ pfu per cornea. As indicated above, at this dose of virus ACKR2^-/-^ mice had more severe disease with higher levels of leukocyte infiltration than WT mice (**Figures 1A** and **2B**). Numerous pro-inflammatory CC chemokines and cytokines including IL-6, and IFN-γ which are important mediators of disease in HSK can induce upregulation of ACKR2 (8, 22, 36, 37). HSK is known to be modulated not only by CC chemokines [reviewed in ref (38)], the selective ligands for ACKR2, but is driven in large part by the chemokine CXCL10 (39–42). This raises a question about how ACKR2 could influence HSK pathogenesis. However, recently it has been shown that ACKR2 binds CXCL10 (43) and thus offers an explanation for the data showing prolonged HSK disease in ACKR2 deficient mice (**Figure 1A**). An alternative mechanism of action by ACKR2 involving impaired DC migration and antigen presentation to T cells is also possible. In previous work, despite reduced numbers of CD207^+^EPCAM^+^ migratory DCs detected in the DLNs of ACKR2^-/-^ mice at day 3 post immunization with antigen, deficiency of ACKR2 did not impair T-cell priming and subsequent development of experimental autoimmune encephalomyelitis (30). This is in agreement with data reported here where expression of co-stimulatory molecules CD40 and CD86 in CD11c^+^ DCs were reduced in ACKR2^-/-^ mice at day 3 p.i. compared to WT mice. However, although DC function in, if not migration to, the eye-draining lymph node might be reduced in ACKR2 deficient HSK mice, there was little evidence of reduced T cell priming since CD4^+^Tbet^+^ and CD4^+^RORγt+ cell populations were equally expanded in ACKR2 deficient and WT mice (**Figure 5C**). Furthermore, the similar rate of viral clearance (**Figure 3**) indicated that lack of ACKR2 on NK cells (44) had no significant role to play.

Importantly, ACKR2 deficiency affected HSK-induced corneal angiogenesis, particularly involving blood vessel formation, but also lymphatics (**Figure 4**). Both lymphangiogenesis and vasculogenesis developed progressively at the same rate during the initial inflammatory response (day 1-7 p.i.) while the enhanced angiogenic response in ACKR2 deficient mice was a relatively late occurrence (14 days p.i.). This appeared to be associated with the persisting inflammation, particularly with CD11b^+^ macrophages which were selectively elevated in numbers at day 7 p.i. (**Figure 5A**). Lymphangiogenesis is a central event in the pathogenesis of HSK (45) and ACKR2 may be required to regulate this response to some extent. In contrast, a more significant effect in this study related to vasculogenesis. ACKR2 is known to restrain angiogenesis in Kaposi’s sarcoma *via* inhibition of pro-angiogenic macrophages (46, 47). However, there may be a more direct effect of ACKR2 on blood vessels. ACKR2 has recently been reported to be expressed in alveolar blood vessels in the lung, predominantly in small capillaries (13). Both small blood vessel angiogenesis and lymphangiogenesis in the cornea have been shown to be under the control of various VEGF isoforms, particularly VEGF-A, produced by pro-inflammatory macrophages [reviewed in ref (48)]. However, it is possible that the upregulation of ACKR2 in HSK observed here (**Figure 1C**) is a tissue vascular response directed towards preventing excessive vascularization. In terms of HSV-1 induced corneal lymphangiogenesis, previous studies have shown that macrophages are not required for corneal lymphangiogenesis at least in the early stage after HSV-1 infection as depletion of corneal macrophages does not affect HSV-1 induced corneal lymphangiogenesis, but identified VEGF-A produced by infected corneal epithelial cells as the main driven factor (49). Furthermore, we have reported previously that in the ACKR2^-/-^ mice, there was accelerated corneal lymphangiogenesis after corneal syngeneic grafting which is likely promoted by increased numbers of infiltrating single Lyve-1^+^ cells in the cornea (26). Interestingly, this discrete Lyve-1^+^ population was not observed in corneas after HSV-1 infection (**Figure 4C**). Thus, despite the possibility of corneal tissue expression of ACKR2, it is more likely that in the context of HSK, ACKR2 limits corneal angiogenesis indirectly *via* its scavenging effect on CC chemokines and macrophages.

Taken together, we show here that ACKR2 is one of several proteins which contribute to recruitment of immune cells in HSK and most likely does so through its decoy role. The lack of ACKR2 does not affect viral elimination but impacts subsequent corneal neovascularization beyond viral clearance. However, this fine-tuning role of ACKR2 is lost when there is severe inflammation.

## Materials and methods

### Animals

The ACKR2^-/-^ mice were bred and maintained on C57BL/6 background. WT littermates were used in all experiments as control mice. Sex-matched 6-8 weeks old mice were used in all experiments. All animals were bred and housed under standard pathogen free conditions at the Medical Research Facility, University of Aberdeen, UK. All animal work was performed in accordance with guidelines of the Association for Research in Vision and Ophthalmology (ARVO) Statement for the use of animals in ophthalmic and vision research and the Animals (Scientific Procedures) Act 1986.

### Corneal HSV-1 infection and clinical evaluation

KOS strain HSV-1 virus (kindly provided by Professor Robert L. Hendricks, University of Pittsburgh, USA) was propagated and purified using Vero cells (cat. 84113001, ECCC, UK). For corneal HSV-1 infection, ACKR2^-/-^ and WT mice were anaesthetized, and right eye corneas scarified with a 25G needle in a 25 x 25 grid pattern followed by application of HSV-1 virus in concentration of 5.4 x 10^5^ pfu or 6.0 x 10^6^ pfu to each cornea. Infected corneas were evaluated using an operating microscope and HSK graded by severity of corneal opacity in a scale of 1 to 4 as described previously (50).

### qPCR analysis

Whole RNA was extracted from cornea samples using the RNeasy micro kit (Qiagen), performing gDNA elimination using the RNase free DNase Set (Qiagen). RNA was then reverse transcribed into cDNA using the High-Capacity RNA-to-cDNA™ Kit (Applied Biosystems). cDNA was used as template for qPCR to determine the expression level of ACKR2 using PerfeCTa^®^ SYBR^®^ Green FastMix ROX (Quanta Biosciences) and the Quant Studio 7Flex (Applied Biosystems). Expression of ACKR2 was calculated using a standard curve and results were normalized to the expression of the housekeeping gene TBP. The following primers were used for ACKR2 and housekeeping gene TBP : ACKR2_s:5’-TTCTCCCACTGCTGCTTCAC-3’;ACKR2_as:5’-TGCCATCTCAACATCACAGA-3’;TBP_s:5’-TGCTGTTGGTGATTGTTGGT-3’;TBP_as:5’-AACTGGCTTGTGTGGGAAAG-3’.

### Corneal whole mounts

Corneal whole mounts from naïve and infected ACKR2^-/-^ and WT mice were prepared and stained as previously described with modifications (51). Briefly, the anterior segment of the eye (cornea, corneal limbus, iris and lens) was excised in one piece and immediately fixed in 4% paraformaldehyde for 30 min at 4˚C. Corneas were then separated from the lens and iris and washed with PBS followed by incubation with methanol for 20 min at room temperature. Tissues were washed, blocked with 10% normal mouse serum and incubated overnight with rat anti-mouse CD31 (550274, BD Bioscience, USA) and rabbit anti-mouse Lyve-1 (ab14917, Abcam, UK) antibodies diluted in PBS-BGEN (3% BSA, 0.25% gelatine, 5mM EDTA and 0.025% IGEPAL CA-630 equivalent to Nonidet-P40) at 4°C. Tissues were washed again in PBS and stained with secondary antibodies Alexa Fluor 555 goat anti-rat IgG (A21434, Invitrogen, USA) and Alexa Fluor 488 goat anti-rabbit IgG (A11070, Invitrogen, USA) diluted in PBS-BGEN for 2 h at room temperature. The corneas were then washed and mounted as single specimens on slides with Hydromount (HS-106, National Diagnostics, GA, USA). Corneal whole mounts were imaged with a Zeiss slide scanner (Zeiss Axio Scan.Z1, Zeiss, Jena, Germany). Images were analyzed with the software Volocity (PerkinElmer, MA, USA) for blood and lymphatic vessel coverage, which was determined as the area between the outer limbal vessel arcade and the junction of the innermost end of newly formed vessels (**Figure 4A**). Lymphangiogenesis was quantified in terms of lymphatic sprouts and loops using ImageJ software (National Institute of Health, USA) and the plug-in Lymphatic Vessel Analysis Protocol (LVAP) as described previously (52).

### Flow cytometry

Lymph nodes were processed into single cell suspensions using a 70 μm cell strainer. Corneas were excised and incubated with 1 mg/ml collagenase D (11088866001, Roche, Switzerland) for 1 h at 37°C to obtain single cell suspensions. Cells were incubated with Fc block (CD16/CD32) for 15 min at 4°C, followed by incubation with directly conjugated monoclonal antibodies (mAb) to CD3 (145-2C11), CD4 (GK1.5), CD45 (30-F11), CD11b (M1/70), CD11c (HL3), Gr-1 (RB6-8C5), CD40 (3/23) and CD86 (GL1) (all from BD Biosciences, USA) for 30 min at 4°C. For staining of intracellular transcription factor, cells were incubated with Cytofix/Cytoperm (BD Biosciences, USA) for 20 min at 4°C followed by staining with directly conjugated, T-bet (4B10, eBioscience, UK) and RORγt (Q31-378, BD Biosciences, USA) for 30 min at 4°C. Thereafter, the cells were washed and data acquired with a BD LSRII Flow Cytometer (BD Biosciences, USA). Data analysis was performed using the FlowJo software (Miltenyi Biotec, Germany). For calculation of absolute cell numbers, the percentage cell population was multiplied by total cell count.

### Viral detection in tear film

Corneal tear film samples were collected from infected ACKR2^-/-^ and WT mice at day 1, 3, 5 and 7 post corneal infection for standard plaque assay as previously described (53, 54). Briefly, foam tipped applicators soaked in DMEM (high glucose) supplemented with 1% penicillin and streptomycin were used to swab infected corneas and stored in DMEM at -80˚C. For standard plaque assay, samples were defrosted, vortexed and 300μl of virus dilutions were incubated on Vero cell monolayers in 6 well plates for 1 h initially at 37°C and 5% CO_2_ with gentle rocking every 15 min. The cells were then overlaid with complete DMEM containing 2% normal human serum (2931149, MP Biomedicals, CA, USA) and incubated for 3 days at 37°C and 5% CO_2_. Giemsa stain was then used to stain Vero cells and plaques formed were counted.

### Statistical analysis

All data were analyzed with GraphPad Prism (GraphPad Software, USA). Clinical scores of HSK were compared using Mann-Whitney *U* test. Unpaired student’s *t*-test and One-way ANOVA with Bonferroni post-test were used for parametric data and Mann-Whitney *U* test was used for non-parametric data. Statistical significance was considered where *p*<0.05 for all experiments.

## Data availability statement

The original contributions presented in the study are included in the article. Further inquiries can be directed to the corresponding author.

## Ethics statement

The animal study was reviewed and approved by Ethical Review Committee, University of Aberdeen, United Kingdom.

## Author contributions

JVF, GJG, and LK contributed to the design of the study. TY, FS and MC performed the experiments. TY, JVF, LK, MC and GJG contributed to data analysis and interpretation. TY, JVF, and LK wrote the manuscript. All authors contributed to the article and approved the submitted version.

## Funding

This work was funded by Fight for Sight, The Eye Charity (CSO project grant award: 3031-3032). The Development Trust of the University of Aberdeen (Saving Sight in Grampian) (Grant No. RG-12663 and RG-14251). The Wellcome Trust Investigator Award (Grant No. 217093/Z/19/Z) and an MRC Programme Grant (Grant No. MRV0109721). The National Natural Science Foundation for Young Scholars of China (Grant No. 81900833).

## Acknowledgments

We thank the Iain Fraser Flow Cytometry core facility, and the Microscopy and Histology core facility of the University of Aberdeen.

## Conflict of interest

The authors declare that the research was conducted in the absence of any commercial or financial relationships that could be construed as a potential conflict of interest.

## Publisher’s note

All claims expressed in this article are solely those of the authors and do not necessarily represent those of their affiliated organizations, or those of the publisher, the editors and the reviewers. Any product that may be evaluated in this article, or claim that may be made by its manufacturer, is not guaranteed or endorsed by the publisher.
